# Incidence of Chorioamnionitis in Patients With Meconium-stained Amniotic Fluid

**DOI:** 10.1155/S1064744995000032

**Published:** 1995

**Authors:** Shelley Chapman, Patrick Duff

**Affiliations:** Division of Maternal-Fetal Medicine University of Florida College of Medicine P.O. Box 100294 Gainesville FL 32610-0294 USA

## Abstract

*Objective:* The goal of this study was to determine if meconium staining of the amniotic fluid (MSAF) is a marker for chorioamnionitis.

*Methods:* In a retrospective, case-control investigation, we studied 100 patients with MSAF. Each patient was matched with a control who delivered during the same period but did not have MSAF. Subjects and controls were matched for age, parity, gestational age, mode of delivery, duration of rupture of membranes (ROM), length of internal monitoring, and number of examinations before and after ROM. The incidence of chorioamnionitis in controls and study patients was compared. The diagnosis of chorioamnionitis was based on clinical examination.

*Results:* Thirteen of the 200 patients [6.5%, 95% confidence interval (CI), 2.5–10.5%] developed chorioamnionitis. Of the 100 women with MSAF, 10 (10%, 95% CI, 4–16) were infected compared with only 3 controls (3%, 95% CI, 0–6, *P* = 0.04). The odds ratio (OR) for this comparison was 3.3, and the 95% CI was 1.02–10.63.

*Conclusions:* MSAF is associated with an increased frequency of chorioamnionitis. Several factors could explain this association. Infection may cause fetal stress, leading to the release of meconium. MSAF may enhance the growth of bacteria by providing a rich medium of essential nutrients or growth stimulants. MSAF also may impair the host immune system so that chemotaxis or phagocytosis is diminished, thus allowing accelerated growth of microorganisms.


Infectious Diseases in Obstetrics and Gynecology 2:210–212 (1995)
© 1995 Wiley-Liss, Inc.

### Incidence of Chorioamnionitis in Patients With Meconium-Stained Amniotic Fluid

**Shelley Chapman and Patrick Duff**

*Division of Maternal-Fetal Medicine, University of Florida College of Medicine, Gainesville, FL*

ABSTRACT

***Objective:*** **The goal of this study was to determine if meconium staining of the amniotic fluid (MSAF) is a marker for chorioamnionitis.**

***Methods:*** **In a retrospective, case-control investigation, we studied 100 patients with MSAF. Each patient was matched with a control who delivered during the same period but did not have MSAF. Subjects and controls were matched for age, parity, gestational age, mode of delivery, duration of rupture of membranes (ROM), length of internal monitoring, and number of examinations before and after ROM. The incidence of chorioamnionitis in controls and study patients was compared. The diagnosis of chorioamnionitis was based on clinical examination.**

***Results:*** **Thirteen of the 200 patients [6.5%, 95% confidence interval (CI), 2.5–10.5%] developed chorioamnionitis. Of the 100 women with MSAF, 10 (10%, 95% CI, 4–16) were infected compared with only 3 controls (3%, 95% CI, 0–6, $P = 0.04$). The odds ratio (OR) for this comparison was 3.3, and the 95% CI was 1.02–10.63.**

***Conclusions:*** **MSAF is associated with an increased frequency of chorioamnionitis. Several factors could explain this association. Infection may cause fetal stress, leading to the release of meconium. MSAF may enhance the growth of bacteria by providing a rich medium of essential nutrients or growth stimulants. MSAF also may impair the host immune system so that chemotaxis or phagocytosis is diminished, thus allowing accelerated growth of microorganisms.**
© 1995 Wiley-Liss, Inc.

KEY WORDS

Intraamniotic infection, meconium staining, amniotic fluid, pregnancy

Approximately 0.5–2% of all term pregnancies are complicated by chorioamnionitis.[1] This condition is associated with a higher frequency of cesarean delivery, prolonged maternal febrile morbidity, and an increased rate of neonatal infection, all of which result in prolonged hospitalization and expense for mother and neonate. Several important risk factors for chorioamnionitis have been identified, including the length of labor, duration of rupture of membranes (ROM), duration of internal monitoring, socioeconomic status of patient, preexisting genital-tract infection, and the number of vaginal examinations. The presence of meconium-stained amniotic fluid (MSAF) may be a sign of fetal stress and is associated with the possibility of meconium aspiration syndrome, but its association with chorioamnionitis has not been precisely defined.[2–5] We undertook the following case-control study to determine if term patients with MSAF had an increased risk of chorioamnionitis.

### MATERIALS AND METHODS

In a retrospective, case-control investigation, we studied 200 pregnant women with singleton term pregnancies from the obstetrics service at Shands Hospital, University of Florida, during the period

**Address correspondence/reprint requests to Dr. Patrick Duff, Division of Maternal-Fetal Medicine, University of Florida College of Medicine, P.O. Box 100294, Gainesville, FL 32610-0294.**

Received August 1, 1994

Accepted October 21, 1994

from January 1991 to May 1993. The patients were predominantly from low socioeconomic backgrounds. One hundred *asymptomatic* women with MSAF were identified using our computerized perinatal database. Each of these women was matched to a control who delivered during the same period but who did not have MSAF. Possible controls were initially identified by a review of the labor and delivery birth log and then confirmed by an assessment of the complete hospital record. Controls were matched to cases for demographic and clinical variables recognized as risk factors for chorioamnionitis.[6,7] The matching variables included age (±3 years), parity (nulliparous, 1–2, 3–5, >5), mode of delivery (vaginal vs. cesarean), length of ROM (<6, 6–12, 13–18, >18 h), length of internal monitoring (0–6, 7–12, >12 h), and number of pelvic examinations both before and after ROM (<4, 4–6, >6).

Patients who presented to the labor-and-delivery suite with overt infection or who were receiving antibiotics at the time of admission were excluded from the study and control groups. Patients were also excluded if they had abnormal presentations.

The diagnosis of chorioamnionitis was based on all of the following clinical criteria: maternal temperature >38.0°C, fetal or maternal tachycardia, and uterine tenderness in the absence of any other localizing signs of infection. The patients with chorioamnionitis were treated with intravenous (IV) antibiotics, usually ampicillin plus gentamicin, as soon as the diagnosis was established, unless delivery was imminent. In the latter case, antibiotics were administered after the infant's umbilical cord was clamped. Clinicians caring for the patients were unaware of the intent of this investigation. At the time of the study, MSAF was not considered an indication for microbiologic assessment of the amniotic flud or empiric antibiotic treatment.

The differences between the study and control groups were compared using the uncorrected chi-squared test. Ninety-five percent confidence intervals (CI) and odds ratios (OR) were also calculated, when appropriate. $P < 0.05$ was considered statistically significant.

### RESULTS

Thirteen of 200 patients (6.5%, 95% CI, 2.5–10.5) developed chorioamnionitis. Eleven (85%) of these patients were younger than 25 years and 10 (77%) were nulliparous. All had ROM for >6 h and 12 (92%) had internal monitoring >6 h. All had >4 vaginal examinations after ROM.

Chorioamnionitis was significantly more common in patients with MSAF than in patients with clear fluid. Of the 100 women with MSAF, 10 (10%, 95% CI, 4–16) were infected compared with only 3 controls (3%, 95% CI, 0–6, $P = 0.04$). The OR for this comparison was 3.3, and the 95% CI for the OR was 1.02–10.63.

### DISCUSSION

The results of this investigation demonstrate a significant increase in the incidence of chorioamnionitis in term patients with MSAF (10%) compared with carefully matched controls without MSAF (3%). In considering the significance of this finding, we acknowledge that our study has potential shortcomings. First, the study design was retrospective. Second, we did not routinely culture either the lower genital tract or amniotic fluid in cases or controls. Therefore, there is a possibility that, despite careful matching for clinical variables, the 2 groups may have differed with respect to frequency of colonization by genital-tract pathogens. Third, our overall frequency of chorioamnionitis was relatively high compared with other reports in the literature. This finding is most likely due to the high prevalence of group B streptococcal colonization and bacterial vaginosis in our population. In previous investigations, we reported the prevalence of these 2 infections to be 26% and 30%, respectively.[8,9] Both of these infections are recognized as important independent risk factors for chorioamnionitis.

Despite the caveats noted above, our observations are supported by a previous study by Wen et al.,[10] who found an 8% incidence of chorioamnionitis in term patients with MSAF compared with 2% in patients without MSAF. This study predominantly consisted of an African-American and Mexican-American population. Although the authors did not specifically match subjects and controls for age, parity, gestational age, mode of delivery, length of ROM, duration of fetal monitoring, and number of pelvic examinations, the groups were relatively comparable with respect to these variables.

Previous reports by Romero et al.[5] and Markovitch and coworkers[11] also identified MSAF as a risk factor for intraamniotic infection in patients having preterm delivery. Romero et al.[5] studied 30 patients with preterm labor and MSAF, detected by transabdominal amniocentesis, and 677 preterm patients with clear amniotic fluid. They reported a significantly higher rate of positive amniotic-fluid cultures in patients with MSAF compared with patients with clear fluid (33% vs. 11%, $P = 0.001$). Markovitch et al.[11] evaluated 89 patients with premature delivery and MSAF and an equal number with premature delivery and clear amniotic fluid. The prevalence of clinical chorioamnionitis was higher in patients with MSAF than in patients with clear fluid (6% vs. 0%, $P = 0.03$). In addition, they found that histologic chorioamnionitis was significantly higher in patients with MSAF (11.2% vs. 0%, $P = 0.03$).

These data plus our own findings suggest that there is an association between MSAF and chorioamnionitis. Two explanations for this association seem plausible. First, microorganisms swallowed by the fetus may induce gastrointestinal motility and meconium passage. Investigators studying the association of intraamniotic infection with *Listeria monocytogenes* and MSAF proposed that the ingestion of infected amniotic fluid leads to fetal enteritis with subsequent meconium passage.[12] Although *L. monocytogenes* is not a common pathogen in term patients with chorioamnionitis, Romero et al.[13] have demonstrated that bacterial endotoxin is more commonly found in MSAF than in clear amniotic fluid (19.3% vs. 3.4%, $P < 0.001$). Therefore, perhaps bacteria or endotoxins present in amniotic fluid cause meconium passage through a local effect on gastrointestinal activity.

A second explanation is that meconium may enhance the growth of bacteria in amniotic fluid or impair host immunity, thus tipping the balance in the host's battle against infection. Florman and Teubner[3] previously demonstrated that meconium enhanced the growth of *Escherichia coli, L. monocytogenes,* and *Staphylococcus aureus* in vitro. More recently, other investigators[14] have suggested that meconium may alter zinc-to-phosphorus ratios in amniotic fluid and thus enhance bacterial growth and decrease host defenses.

We conclude that, compared with matched term women with clear amniotic fluid, women in our institution with MSAF are more likely to develop chorioamnionitis. Accordingly, we suggest that asymptomatic women with MSAF should be observed carefully for early signs of clinical infection.

### REFERENCES

1. Isada NB, Grossman JH: Perinatal infections. In Gabbe SG, Niebyl JR, Simpson JL (eds): Obstetrics. Normal and Problem Pregnancies. 2nd ed. New York: Churchill Livingstone, p 1276, 1991.
2. Miller FC, Sacks DA, Yets SY, et al.: Significance of meconium during labor. Am J Obstet Gynecol 122:573–580, 1974.
3. Florman AL, Teubner D: Enhancement of bacterial growth in amniotic fluid by meconium. J Pediatr 74: 111–114, 1969.
4. Blot P, Milliez J, Breart G, et al.: Fetal tachycardia and meconium staining: A sign of fetal infection. Int J Gynaecol Obstet 21:189–194, 1983.
5. Romero R, Hanaoka S, Mazor M, et al.: Meconium-stained amniotic fluid: A risk factor for microbial invasion of the amniotic cavity. Am J Obstet Gynecol 164: 859–862, 1991.
6. Soper DE, Mayhall CG, Dalton HP: Risk factors for intraamniotic infection: A prospective epidemiologic study. Am J Obstet Gynecol 161:562–568, 1989.
7. Newton ER, Prihoda TJ, Gibbs RS: Logistic regression analysis of risk factors for intraamniotic infection. Obstet Gynecol 73:571–575, 1989.
8. Yancey MK, Duff P, Clark P, Kurtzer T, Frentzen BH, Kubilis P: Peripartum infection associated with vaginal group B streptococcal colonization. Obstet Gynecol 84: 816–820, 1994.
9. Clark P, Kurtzer T, Duff P: The role of bacterial vaginosis in peripartum infections. Infect Dis Obstet Gynecol (in press).
10. Wen TS, Eriksen NL, Blanco JD, et al.: Association of clinical intraamniotic infection and meconium. Am J Perinatol 10:438–440, 1993.
11. Markovitch O, Mazor M, Shoham-Vardi I, et al.: Meconium stained amniotic fluid is associated with maternal infectious morbidity in preterm delivery. Acta Obstet Gynaecol Scand 72:538–542, 1993.
12. Halliday HL, Hirata T: Perinatal listeriosis. A review of twelve patients. Am J Obstet Gynecol 133:405–410, 1979.
13. Romero R, Mazor M, Sepulveda W, et al.: Is bacterial endotoxin a cause of meconium passage in utero? Society of Perinatal Obstetricians—12th Annual Meeting, February 3–8, 1992, Orlando, Abstract 5.
14. Hoskins IA, Hemming VG, Johnson TR, Winkel CA: Effects of alterations of zinc-to-phosphorus ratios and meconium content on group B streptococcus growth in human amniotic fluid in vitro. Am J Obstet Gynecol 157:770–773, 1987.
